# Genome-Wide Association Studies and Runs of Homozygosity to Identify Reproduction-Related Genes in Yorkshire Pig Population

**DOI:** 10.3390/genes14122133

**Published:** 2023-11-27

**Authors:** Lige Zhang, Songyuan Zhang, Meng Yuan, Fengting Zhan, Mingkun Song, Peng Shang, Feng Yang, Xiuling Li, Ruimin Qiao, Xuelei Han, Xinjian Li, Meiying Fang, Kejun Wang

**Affiliations:** 1 College of Animal Science and Technology, Henan Agricultural University, Zhengzhou 450002, China; hsnimitz@163.com (L.Z.); songyuanzhang2022@163.com (S.Z.); 19513113121@163.com (M.Y.); 17837161862@163.com (F.Z.); m18625951565@163.com (M.S.); chinafy6868@163.com (F.Y.); xiulingli@henau.edu.cn (X.L.); qrm480@163.com (R.Q.); hxl014@126.com (X.H.); lxjlongfei@163.com (X.L.); 2 Animal Science College, Tibet Agriculture and Animal Husbandry University, Linzhi 860000, China; nemoshpmh@126.com; 3 Department of Animal Genetics and Breeding, National Engineering Laboratory for Animal Breeding, MOA Laboratory of Animal Genetics and Breeding, College of Animal Science and Technology, China Agricultural University, Beijing 100193, China

**Keywords:** pig, reproductive trait, GWAS, ROH island

## Abstract

Reproductive traits hold considerable economic importance in pig breeding and production. However, candidate genes underpinning the reproductive traits are still poorly identified. In the present study, we executed a genome-wide association study (GWAS) and runs of homozygosity (ROH) analysis using the PorcineSNP50 BeadChip array for 585 Yorkshire pigs. Results from the GWAS identified two genome-wide significant and eighteen suggestive significant single nucleotide polymorphisms (SNPs) associated with seven reproductive traits. Furthermore, we identified candidate genes, including *ELMO1*, *AOAH*, *INSIG2*, *NUP205*, *LYPLAL1*, *RPL34*, *LIPH*, *RNF7*, *GRK7*, *ETV5*, *FYN*, and *SLC30A5*, which were chosen due to adjoining significant SNPs and their functions in immunity, fertilization, embryonic development, and sperm quality. Several genes were found in ROH islands associated with spermatozoa, development of the fetus, mature eggs, and litter size, including *INSL6*, *TAF4B*, *E2F7*, *RTL1*, *CDKN1C*, and *GDF9*. This study will provide insight into the genetic basis for pig reproductive traits, facilitating reproduction improvement using the marker-based selection methods.

## 1. Introduction

Enhancing the fertility of pigs has been a perennially intriguing topic in the pig industry [1,2]. Molecular breeding, which involves the identification of SNPs and candidate genes associated with reproduction, has proven to be an efficacious approach to augment reproductive capacity [3]. However, the heritability of litter size is generally low [4]. Litter size directly reflects the productivity of pigs per sow per year [5]. Many researchers have dedicated their efforts to identifying SNPs and candidate genes that are associated with litter size [6,7,8], but there is still a long way to go to improve pig reproduction.

A genome-wide association study (GWAS) is a new method being used to accelerate the speed of progress in pig breeding [9]. The GWAS has been used to find new single nucleotide polymorphisms (SNPs) and to detect candidate genes for important economic traits in animals [10]. Improving reproductive traits including the number of teats [11], litter size [12], TNB, NBA [13], etc., is a great and helpful way to increase pig performance and economic benefits. Of course, decreasing the number of dead pigs [14], fetuses [15], and so on also cuts down the pecuniary losses. In this research, we collected more detailed pig litter records to perform a GWAS in a Yorkshire sow population and discover new SNPs and candidate genes from these traits.

Runs of homozygosity (ROH) are long, consecutive homozygous fragments in the genome [16] and are used to calculate the extent of identical haplotypes which are inherited from the parents [17], especially in inbred pigs. ROH are a crucial genome feature that provide a valuable reference for genome structure analysis. In animal genetics, the occurrence of homozygous genome segments is influenced by intensive selection, population history, and level of consanguinity [18]. Therefore, it is recognized that ROH hotspots exhibit a non-random distribution throughout the genome, and ROH islands are shown to be distributed and shared in individuals, which is likely a result of selection events [19,20]. In recent years, ROH analysis has been widely utilized to assess genomic inbreeding and detect selection signatures in various livestock populations [21], so ROH can be used as a way to detect signal selection in populations.

The present investigation endeavors to employ a GWAS to pinpoint noteworthy SNPs or genes linked to reproductive traits in Yorkshire pigs, a breed renowned for its superior reproductive performance, and to identify the genomic regions that have undergone selection for reproductivity via ROH analysis. Identifying reproduction-related genes in pigs can give perspective on the underlying molecular mechanisms of their reproductive capacity. The identification of genes associated with pig reproduction can provide insights into the underlying molecular mechanisms, and these results would help us to improve the understanding of reproductive capacity and performance in the breeding of pigs.

## 2. Materials and Methods

### 2.1. Sample Collection

A total of 585 female American Yorkshire pigs were obtained from the Beijing Swine Center in Beijing, China. These pigs were provided with a uniform diet and subjected to a standardized raising and management protocol, with ad libitum feeding. Throughout the entire duration of the experiment, none of the pigs experienced any physical or psychological distress, nor did they exhibit any signs of illness. The collection of samples was conducted following a specific procedure, which involved cleaning the area with an alcohol-soaked cotton ball (with a concentration of 75%) and subsequently cutting a small piece from the pig’s ear. Records of seven reproductive traits, including total number born (TNB), number born alive (NBA), number born strong (STRONG), number born weak (WEAK), number of born freak (FREAK), number of stillborn (DEAD), and number of mummified (MUMMY), were collected by professional breeders. The protocol for collecting ear tissue was approved by the Animal Welfare Committee of China Agricultural University (approval number XK257).

### 2.2. Genotyping and Quality Control

DNA was extracted from ear tissue using the phenol–chloroform method [22], and its quality and quantity were assessed using a NanoDrop 2000 spectrophotometer (Thermo Fisher Scientific Inc., Waltham, MA, USA). Genotyping was carried out using the GeneSeek-Neogencine SNP60 BeadChip (Beijing Kangpusen Biological Technology Co., Ltd., Beijing, China). For other specific processing steps of genotyping we referred to previous articles [23]. In order to minimize false-positive associations resulting from genotyping errors, we used PLINK 1.9 (http://www.cog-genomics.org/plink/1.9 accessed on 14 May 2023) to perform quality control for 50K SNP chip data. We retained SNPs with a genotyping call rate greater than 99% and a minor allele frequency (MAF) of ≥1%. All autosomes were kept, and sex chromosomes were removed. After a serious filtration step, a total of 49,284 SNPs were retained to perform association analysis with reproductive traits.

### 2.3. Phenotypic Correlation Analysis

To calculate the correlation between the seven reproductive-related traits collected from Yorkshire pigs, we carried out correlation analysis. Correlation analysis of the seven reproductive traits of pigs was calculated using Spearman’s (r_P_) and Pearson’s (r_S_) correlation to ensure the reliability of the correlation results. The gcorrplot package version 0.1.4.1 (https://github.com/taiyun/corrplot accessed on 17 May 2023) was used for phenotypic correlation analysis, and ggplot2 version 3.4.4 (https://github.com/tidyverse/ggplot2 accessed on 18 May 2023) was used for visualization. The results of the association analysis retain two significant digits.

### 2.4. Principal Component Analysis (PCA) and Relationship Analysis

We then carried out population structure analysis, including principal component analysis and kinship analysis, to determine whether the population stratified. If the population stratified, the results of the GWAS analysis would have a great false-positive phenomenon. The results of the principal component analysis can be added to the GWAS analysis as a covariate to reduce false-positive results. Principal component analysis (PCA) was estimated using SNP data with GCTA version 1.94.0 (https://yanglab.westlake.edu.cn/software/gcta/ accessed on 8 May 2023). Then, pairwise kinship was estimated using genome-wide autosomal SNP information with the IBS [24] function in PLINK version 1.9. PCA and kinship visualization used scatterplot3d version 0.3.44 (https://cran.r-project.org/web/packages/scatterplot3d/ accessed on 10 May 2023) and pheatmap version 1.0.12 (https://CRAN.R-project.org/package=pheatmap accessed on 13 May 2023), respectively.

### 2.5. Genome-Wide Association Study

GWAS was performed using R statistical software, version 4.0.2, (https://www.r-project.org/ accessed on 15 September 2020) using the Fixed and random model Circulating Probability Unification (FarmCPU) method [25] in GAPIT, version 3.0 (https://www.maizegenetics.net/gapit accessed on 12 November 2020). The first three principal component analysis results were used as covariates in the GWAS. Bonferroni multiple tests were employed to identify the genome-wide significant (0.05/number of SNPs) and suggestive (1/number of SNPs) SNPs. Significant association between SNPs and reproductive traits was visualized in Manhattan plots and QQ-plots, which were generated using rMVP (https://github.com/xiaolei-lab/rMVP accessed on 23 November 2020) and CMplot (https://cran.r-project.org/web/packages/CMplot/ accessed on 22 October 2020). The functional genes were annotated using pig reference genome version 11.1 (http://asia.ensembl.org/Sus_scrofa/Info/Index accessed on 25 May 2023) in ENSEMBLE (http://asia.ensembl.org/ accessed on 25 May 2023).

### 2.6. Detection of ROH Segments and Common Runs of Homozygosity

The purpose of this part was to detect ROH segments and identify the genomic regions most commonly associated with ROHs, and the percentage occurrence of SNPs in ROH was computed by counting the number of SNPs detected in ROH for all individuals in the population. SNPs in the top 1% of frequency of occurrence were selected as potential ROH islands [26,27,28]. To determine these, we counted the number of SNPs detected in ROH for all individuals in the population and computed the percent occurrence of SNPs in ROH. Using a 50K SNP Chip and the R package detectRUNS (https://cran.r-project.org/web/packages/detectRUNS/index.html accessed on 11 November 2022), we calculated the frequency of occurrence for each SNP. The detectRUNS’s parameters of ROH island detection were as follows: We categorized the ROHs into five groups based on size: 0 to <6 Mb, 6 to <12 Mb, 12 to <24 Mb, 24 to <48 Mb, and ≥48 Mb. Then, we employed a sliding window analysis using a window size of 15 SNPs, with a minimum requirement of 20 homozygous SNPs. In addition, windows with a homozygous threshold of 0.05 were included, with a minimum of 1000 SNPs per kbps. Moreover, we set the maximum distance between two SNPs to 1,000,000 bps and the minimum length of a homozygous run to 250,000 bps. For the SNPs in the top 1% of frequency of occurrence, we also used ENSEMBLE, based on pig reference genome version 11.1, to annotate the functional genes in the ROH island.

## 3. Results and Discussion

### 3.1. Correlation Analysis between Phenotypes

**Figure 1:**
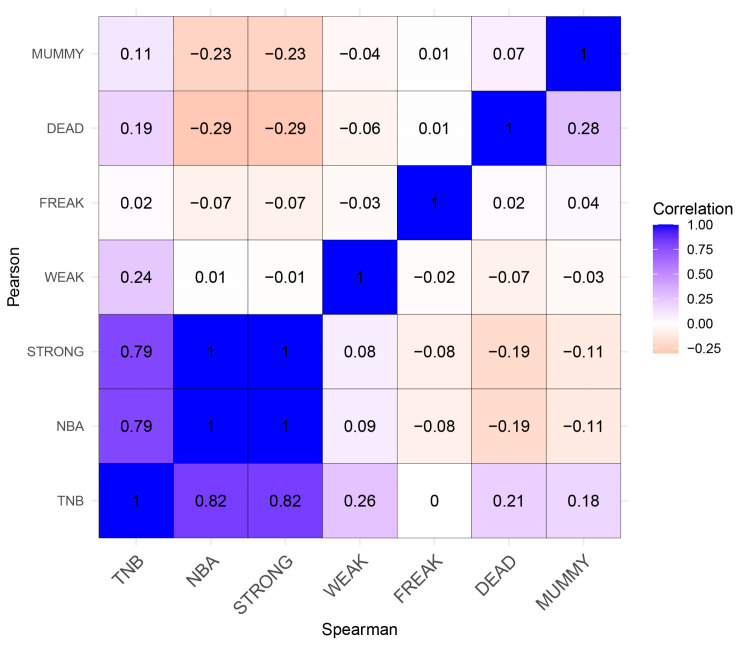
Spearman (lower triangle) and Pearson (upper triangle) correlation coefficient among seven reproductive traits in Yorkshire pigs. In this diagram, positive correlations are depicted using the color blue, while negative correlations are represented by the color red.

In the Yorkshire pig population, we estimated phenotypic association analysis between the TNB, NBA, STRONG, WEAK, FREAK, DEAD, and MUMMY traits, and the results of the correlation analysis are shown in Figure 1. Additionally, the phenotype data of the seven reproductive traits are summarized in Supplementary Table S1.

The two correlations are basically consistent, and the details are as follows: three traits (TNB, NBA, and STRONG) were strongly correlated, ranging from 0.79 to1, among which the STRONG and NBA traits had the strongest positive correlation (1.00), while the TNB trait had a weak-positive correlation. To some extent, this illustrates the close relationship between the STRONG and NBA traits, and this confirms our understanding that as long as we can maximize the improvement of the STRONG trait, we can increase the NBA trait. In addition, genes affecting all three traits may have similar effects, so a gene affecting one trait may affect the other two. The DEAD and MUMMY traits were strongly negatively correlated with the NBA and STRONG traits, ranging from −0.11 to −0.29. The DEAD trait was the most negatively correlated with the NBA and STRONG traits, while the MUMMY trait was strongly correlated with the NBA trait (r_S_: −0.11, r_P_: −0.23); the STRONG trait (r_S_: −0.11, r_P_: −0.23) is second only to the DEAD trait. Therefore, during breeding, attention should be paid to the reduction of the DEAD and MUMMY traits, which are less correlated (r_S_: 0.28, r_P_: 0.07).

### 3.2. PCA and Kinship Analysis

**Figure 2:**
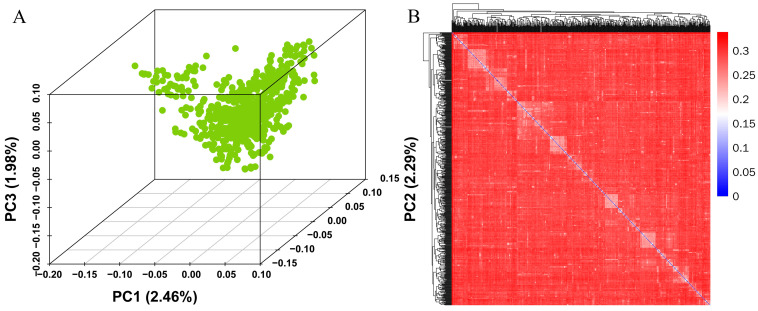
The first three principal components (**A**) and kinship heat map (**B**) of the Yorkshire pig populations.

Population stratification is a significant confounding factor in GWASs, as systematic ancestry differences can lead to false-positive associations [29]. Figure 2A depicts the population stratification of Yorkshire pigs based on PCA; the pig population was not divided into separate clusters of signaling, demonstrating no stratification in the reference population. The results of the principal component analysis (PCA) were consistent with the previous PLINK analysis [23] and did not reveal any significant clustering. The first three PCAs explain the 2.46%, 2.29%, and 1.98% of variation, respectively; about 4% of the variation is explained by the first three PCAs together. In addition, the first three principal components were selected as covariables to eliminate the influence of population stratification on the correlation analysis. The kinship heat map based on the IBS matrix (Figure 2B) also shows us a similar population structure to the PCA results, and there does not appear to be significant population stratification.

Kinship also verified the principal component analysis results, and the two confirmed each other. This suggests that there may be slight false positives in this population, which will be greatly reduced after adding PCA as a covariate to the GWAS calculation.

### 3.3. The Significant SNPs and Genes from GWAS

**Figure 3:**
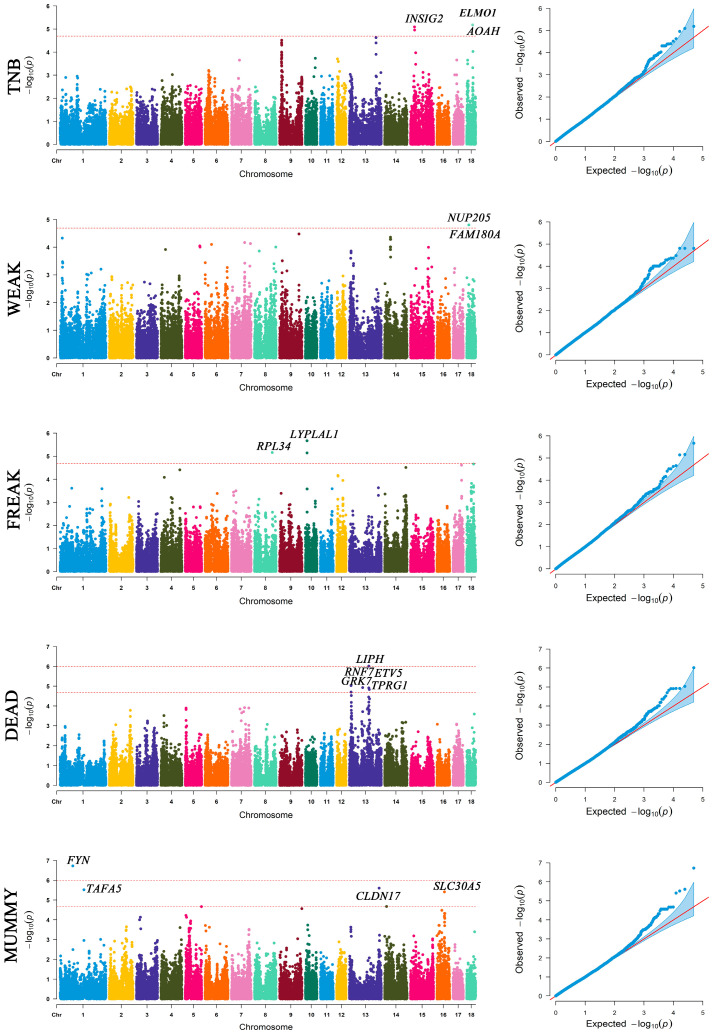
Manhattan plots and quantile–quantile plots of GWAS results using FarmCPU, which confirm the TNB, WEAK, FREAK, DEAD, and MUMMY traits. The suggestive threshold is negative log10 2.03 × 10^−5^, and the genome-wide significant is negative log10 1.01 × 10^−6^. The two red dashed lines represent genome-level significance (up) and chromosomal significance (down).

A total of 2 genome-wide significant and 18 suggestive significant SNPs were identified to be associated with five traits, including TNB, WEAK, FREAK, DEAD, and MUMMY (Figure 3 and Table 1). The QQ-plots for these GWAS results are also reported in Figure 3. No significant association was found between SNPs and the number of strong piglets (STRONG) or the number born alive (NBA).

The two genome-wide significant SNPs, ALGA0071870 relating to the DEAD trait (*p* = 9.56 × 10^−7^) and MARC0022221 relating to the MUMMY trait (*p* = 1.88 × 10^−7^), were located within the *LIPH* and *FYN* gene regions. Several studies have shown that *LIPH* is associated with immunity [30,31,32,33], and the presence of infections in pregnant mothers can lead to abnormal fetal development [34]. Therefore, it is speculated that *LIPH* may, to some extent, lead to stillbirths. *FYN* protein kinase has a crucial role in signal transduction after fertilization [35], and the *FYN* gene has been linked to abnormal sperm [36]. Although abnormal sperm and eggs can be fertilized normally, they can lead to abnormal embryo development and early miscarriage [37,38]. Thus, *FYN* may be closely related to the production of mummified fetuses in pigs.

TNB is an important indicator for predicting pig litter performance. We found that the *ELMO1*, *AOAH*, and *INSIG2* genes were associated with the TNB trait. The engulfment and cell motility 1 (*ELMO1*) gene has been demonstrated to play significant roles in the clearance of apoptotic germ cells and spermatogenesis in mice [39], and the elimination of apoptotic germ cells and sperm is an important process to maintain reproductive health and function [40]. It is evident that *ELMO1* likely has an impact on the total litter size of pigs. The *acyloxyacyl hydrolase* (*AOAH*) gene is expressed through AMPs (antimicrobial peptides and proteins) in placental cells, which is a result of a co-operation of leukocytes and cells from early embryonic development [41]. Therefore, *AOAH* may play an important role in early embryonic immunity, which might reduce the invasion of various viruses and germs in the embryo, ensure embryonic development, and thus ensure the survival of the embryo to a certain extent. Two suggestive significant SNPs are located within or near the *INSIG2* gene (Table 1). The *insulin-induced gene 2* (*INSIG2*) gene is involved in cholesterol anabolism and maintaining the homeostasis of cholesterol [42,43,44]. Homeostasis of cholesterol is possibly crucial for the maternal environment and for providing conditions for the normal development of the fetus, since cholesterol is an important component of the placenta and fetal development [45,46,47,48]. According to the above phenotypic correlation, the TNB trait has a strong correlation with the NBA and STRONG traits, and the above genes may also play an important role in the development of the NBA and STRONG traits (Table 2).

The *nucleoporin 205* (*NUP205*) was related to the WEAK trait, and it was related to ensuring normal embryo development [65]. The quality of embryonic development directly affects the growth and development of the fetus [66], so *NUP205* may play a certain role in fetal survival. The *ribosomal protein L34* (*RPL34*) and *lysophospholipase-like 1* (*LYPLAL1*) were also found to be associated with the FREAK trait. Some studies have found that *RPL34* is associated with infertility triggered by sperm quality [67]. Female eggs can bind to abnormal sperm at the time of conception, which ultimately leads to fetal abnormalities, and *RPL34* can serve as an entry point for studying these causes. *LYPLAL1* is related to diet-induced obesity [68], which may make the sow too obese during pregnancy. Obesity in sows can lead to reduced uterine volume and affect embryo development [69,70], resulting in poor fetal malnutrition immunity and susceptibility to illness [71], which may also be the main cause of the increase in weak litters. Therefore, it is speculated that *LYPLAL1* may increase the weight of the pregnant mother and affect fetal development.

The genes found to be associated with the DEAD trait include *the ring finger protein 7* (*RNF7*), *G protein-coupled receptor kinase* (*GRK7*), and *ets variant gene 5* (*ETV5*). The *RNF7* gene, identified as the gene most similar to the expression of *PCOS* in transgender people, may lead to ubiquitination of the androgen receptor and eventually lead to antral follicular growth stagnation [72]. This may affect the mother’s conception and pregnancy, which is not good for the fetus. It has been found that *GRK7* can perform highly effective photo-oxidation of photoactivated visual pigments in cone cells [73]. This may lead to an increase in the body’s biochemical reaction phosphate bond generation and reconstruction of high-energy bonds, increase the body’s energy reserves, and may also provide conditions for the body to produce more fat. This will lead to weight gain in the mother, which is not conducive to maternal pregnancy. Pathways of *ETV5* are deeply related to the *epithelial-to-mesenchymal transition* (*EMT*) process in *endometrial cancer* (*EC*) [74]. *ETV5* plays an important role in many aspects of development including embryonic and perinatal survival, postnatal growth, limb patterning, kidney development, and fertility [75]. Moreover, the loss of *ETV5* reduces the proliferation and *RET* levels of testicular germ cells in newborn mice and leads to abnormalities in the first wave of spermatogenesis [76]. Maternal pregnancy is affected by obesity and abnormal sperm, and these factors can be the leading cause of fetal death.

Regarding the *solute carrier family 30 member 5* (*SLC30A5*) related to the MUMMY trait, *SLC30A5* has a relation to zinc homeostasis [77] and zinc transporters [78]. Zinc is a key element in embryonic development, and if the mother is deficient in zinc during pregnancy, it may cause a variety of complications for the fetus [79]. In addition, relevant studies have shown that enteral zinc supplementation prevents preterm birth and death of fetuses [80]. Therefore, *SLC30A5* may affect the development of the fetus in the mother by affecting the synthesis and transport of zinc. Based on the results of the phenotypic correlation analysis, we observed a significant correlation between the traits FREAK, DEAD, and MUMMY. This suggests that the genes associated with these traits may also contribute to the expression of the other two traits and thus, they should be further investigated. Unexpectedly, four genes, including *FAM180A*, *ENSSSCG00000047980*, *TPRG1*, and *CLDN17*, adjacent to suggestive significant SNPs, have no published research on their biological function.

### 3.4. ROH Detection and Candidate Genes within Runs of Homozygosity Islands

**Figure 4:**
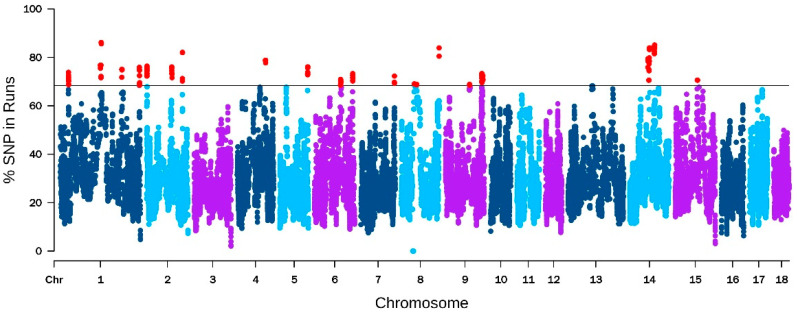
Manhattan plot of the frequency (%) of each SNP in the runs of homozygosity among the total pig population. The solid line represents the 68.38% threshold and the red dots represent the top 1%.

The results of ROH detection show that the number of five kinds of ROH segments were: 141,334 (0–6 Mb), 8573 (6–12 Mb), 3853 (12–24 MB), 1249 (24–48 MB), and 292 (>48 Mb), respectively. The number and proportion of ROH on each chromosome are shown in Supplementary Figure S1. These results show that Yorkshire pigs had more long ROH fragments than in previous research of Chinese native pigs [81], indicating that Yorkshire pigs have a higher inbred coefficient, which may also be one of the reasons for their good performance. Long runs of homozygosity (ROH) can serve as an indicator of recent generation kinship due to the lower probability of interruption recombination events in shorter generations, so the longer ROH fragments suggest a higher likelihood of inbreeding within the population [82]. The percentage of SNPs in ROHs are plotted against the chromosome position in Figure 4. The top 1% of SNPs in the ROH with an over 68.38% occurrence were selected for further analysis. Based on these SNPs, we identified the genomic regions and genes that are most associated with ROH across all individuals. The 12 candidate genes associated with production traits including spermatogenesis, embryonic development, and litter size were identified in the ROH islands.

Six genes related to spermatozoa include *INSL6*, *PSMA8*, *SPATA6*, *TAF4B*, *TPD52L3*, and *HERC4*. *INSL6* ensures sperm motility and is required for the progression of spermatogenesis [49,50]. *PSMA8* is a component of the sperm proteasome, and its deletion reduces proteasome abundance in the testis [51,52]. *SPATA6* is required to connect the sperm head and tail during spermatogenesis [53]. *TAF4B* plays a role in spermatogenesis and oogenesis [54], and it maintains the development of mouse sperm stem cells [83,84]. Some studies indicated a potential role for *TPD52L3* in testis development and spermatogenesis [55]. Moreover, *HERC4* is required for sperm maturation [56]. Improving the quality of sperm can improve the fecundity of sows, and targeting these genes may be important in improving the fecundity of sows.

Next on the list are genes involved in embryonic and fetal development, including *AGBL4*, *AHI1*, *E2F7*, *RTL1*, and *CDKN1C*. *AGBL4* is involved in *KLF4* deglutamylation which promotes *KLF4* proteasome-mediated degradation, thereby negatively regulating embryogenesis [57]. As a positive modulator of classical Wnt signaling, *AHI1* may play a crucial role in ciliary signaling during cerebellum embryonic development [58]. The function of *E2F7* in the extra-embryonic trophoblast is critical in developing the fetus [59,60]. *RTL1* can maintain the fetal and maternal interface and placental development [61]. *CDKN1C* may cause fetal dysplasia and miscarriage [62]. These genes may play an important role in the fecundity of pigs.

Furthermore, *GDF9* is a factor secreted by oocytes, which can promote the development of primordial follicles and regulate the quality and developmental ability of eggs [63,64]. *GDF9* mutation can affect ewe fertility by affecting follicles and oocytes [85]. Previous studies showed that *GDF9* had some connection with the litter size in sheep, and *GDF9* may affect the ovulation rate and litter size in sheep by promoting ovulation [86,87,88]. These results indicate that *GDF9* may be an important gene affecting porcine calving traits.

## 4. Conclusions

In this study, we performed a GWAS and ROH analysis to identify reproduction-related genes with a 50K BeadChip of Yorkshire pigs. We identified a total of 20 candidate SNPs for seven reproductive traits in Yorkshire pigs using the GWAS. Furthermore, the genes, including *ELMO1*, *AOAH*, *INSIG2*, *NUP205*, *LYPLAL1*, *RPL34*, *LIPH*, *RNF7*, *GRK7*, *ETV5*, *FYN*, and *SLC30A5*, identified by these SNPs have a range of functions associated with immunity, sperm, embryonic development, and pregnancy. Moreover, we identified 12 ROH islands containing genes (*INSL6*, *TAF4B*, *E2F7*, *RTL1*, *CDKN1C*, and *GDF9*) with molecular functions involving the spermatozoa, development of the fetus, mature eggs, and litter size. These findings can contribute to the understanding of reproductive performance and enhance future breeding strategies by providing insight for animal husbandry production.

## Figures and Tables

**Table 1:** The genome-wide significant and suggestive SNPs and candidate genes associated with reproductive traits. SNPs surpassing the genome-wide threshold are in the black and bold words, and the other unmarked SNPs reach the suggestive threshold.

Trait	SNP	Ref SNP	SSC	*p* Value	Candidate Genes	Distance (bp)
TNB	ASGA0079603	rs81468948	18	6.49 × 10^−6^	*ELMO1* *AOAH*	Within 343,972
TNB	DRGA0015019	rs81301323	15	8.10 × 10^−6^	*INSIG2*	Within
TNB	ALGA0084417	rs81451913	15	1.10 × 10^−5^	*INSIG2*	203,311
WEAK	ALGA0097098	rs81471717	18	1.57 × 10^−5^	*NUP205*	16,550
WEAK	ASGA0078964	rs81471771	18	1.57 × 10^−5^	*FAM180A*	24,824
WEAK	H3GA0050399	rs81471732	18	1.57 × 10^−5^	*FAM180A*	26,006
FREAK	ALGA0056905	rs81428907	10	2.15 × 10^−6^	*LYPLAL1*	252,956
FREAK	WU_10.2_8_121864282	rs328492172	8	6.98 × 10^−6^	*RPL34*	154,848
FREAK	DRGA0010254	rs81302520	10	7.24 × 10^−6^	*LYPLAL1*	219,860
DEAD	**ALGA0071870**	**rs80920882**	13	9.56 × 10^−7^	*LIPH* *ENSSSCG00000051486*	Within 73,460
DEAD	ASGA0100324	rs81321047	13	9.27 × 10^−6^	*ENSSSCG00000047980*	248,227
DEAD	ASGA0058420	rs80872186	13	1.18 × 10^−5^	*RNF7*	41,373
DEAD	ASGA0058424	rs81447229	13	1.18 × 10^−5^	*GRK7*	Within
DEAD	ASGA0099314	rs81319768	13	1.23 × 10^−5^	*ETV5*	Within
DEAD	MARC0004732	rs81232688	13	1.23 × 10^−5^	*ETV5*	Within
DEAD	SIRI0000314	rs329411938	13	1.47 × 10^−5^	*TPRG1*	26,006
MUMMY	**MARC0022221**	**rs81290729**	1	1.88 × 10^−7^	*FYN*	Within
MUMMY	WU_10.2_13_204127273	rs324169348	13	2.48 × 10^−6^	*CLDN17*	9458
MUMMY	ASGA0004838	rs81348947	1	3.02 × 10^−6^	*TAFA5*	92,417
MUMMY	MARC0028125	rs81223068	16	3.90 × 10^−6^	*SLC30A5*	147,756

SNP: single nucleotide polymorphism; ref SNP: reference SNP identification from the SNP database; SSC: Sus scrofa chromosome.

**Table 2:** The genomic regions and functions of extended homozygosity (ROH islands) identified in Yorkshire pigs.

CHR	Start (bp)	End (bp)	Gene Symbol	SNP %	Function
1	216,792,785	216,820,197	*INSL6*	75.04	Ensures sperm motility [49,50]
6	110,799,065	110,836,937	*PSMA8*	69.91	Component of the sperm proteasome [51,52]
6	163,278,954	163,410,174	*SPATA6*	71.62	Connects the sperm head and tail during spermatogenesis [53]
6	110,855,636	110,972,798	*TAF4B*	69.91	Plays a role in spermatogenesis and oogenesis [54]
1	215,841,463	215,842,606	*TPD52L3*	71.79	Potential role in testis development and spermatogenesis [55]
14	71,126,100	71,269,517	*HERC4*	83.93	Necessary for sperm maturation [56]
6	161,953,495	163,216,282	*AGBL4*	73.33	Regulates embryogenesis [57]
1	28,566,279	28,760,471	*AHI1*	71.79	Plays a crucial role in ciliary signaling during cerebellum embryonic development [58]
5	104,126,924	104,163,582	*E2F7*	76.07	Critical for development of the fetus [59,60]
7	121,706,955	121,720,472	*RTL1*	69.40	Maintains fetal and maternal interface and placental development [61]
2	2,019,391	2,022,092	*CDKN1C*	72.99	Causes fetal dysplasia and miscarriage [62]
2	135,181,494	135,184,451	*GDF9*	82.05	Regulates the quality and developmental ability of eggs [63,64]

Abbreviations: CHR: pig chromosome; Start: start position of the gene; End: end position of the gene; SNP %: the incidence of SNPs in the runs of homozygosity.

## Data Availability

All animals involved in this study were managed according to the instructions of the care and use of experimental animals established. The genotype data for Yorkshire pigs are owned by Henan Agricultural University. Data from the current study are available via https://osf.io/jqtw8/ (accessed on 14 July 2023).

## Supplementary Materials

The following supporting information can be downloaded at: https://www.mdpi.com/article/10.3390/genes14122133/s1, Table S1: Statistical information on seven reproductive traits of Yorkshire pigs; Figure S1: The number and proportion of ROHs on each chromosome.
