# Supplementary material for: Genome-Wide Association Studies and Runs of Homozygosity to Identify Reproduction-Related Genes in Yorkshire Pig Population
Source: Genes (Basel). 2023 Nov 27;14(12):2133. doi: 10.3390/genes14122133 (PMC10742578; doi:10.3390/genes14122133)
Supplement: Supplementary file 1 [file genes-14-02133-s001.zip › Supplementary Table1ú║Descriptive statistics for reproductive traits in Yorkshire pig population.pdf]

Table S1. Descriptive statistics for reproductive traits in Yorkshire pig population.

Phenotype	Number of individuals	Average value	Standard deviation	Median	Minimum	Maximum
**Total number born alive (TNB)**	559	10.42	2.93	11.00	1.00	21.00
**Number born alive (NBA)**	559	9.14	2.95	9.00	0.00	17.00
**Number born strong (STRONG)**	559	9.12	2.96	9.00	0.00	17.00
**Number of born weak (WEAK)**	559	0.42	0.85	0.00	0.00	9.00
**Number of born freak (FREAK)**	559	0.04	0.26	0.00	0.00	3.00
**Number of stillborn (DEAD)**	559	0.68	1.42	0.00	0.00	15.00
**Number of mummified (MUMMY)**	559	0.17	0.94	0.00	0.00	15.00
