# Supplementary figures and images for: Genome-Wide Association Studies and Runs of Homozygosity to Identify Reproduction-Related Genes in Yorkshire Pig Population
Source: Genes (Basel). 2023 Nov 27;14(12):2133. doi: 10.3390/genes14122133 (PMC10742578; doi:10.3390/genes14122133)

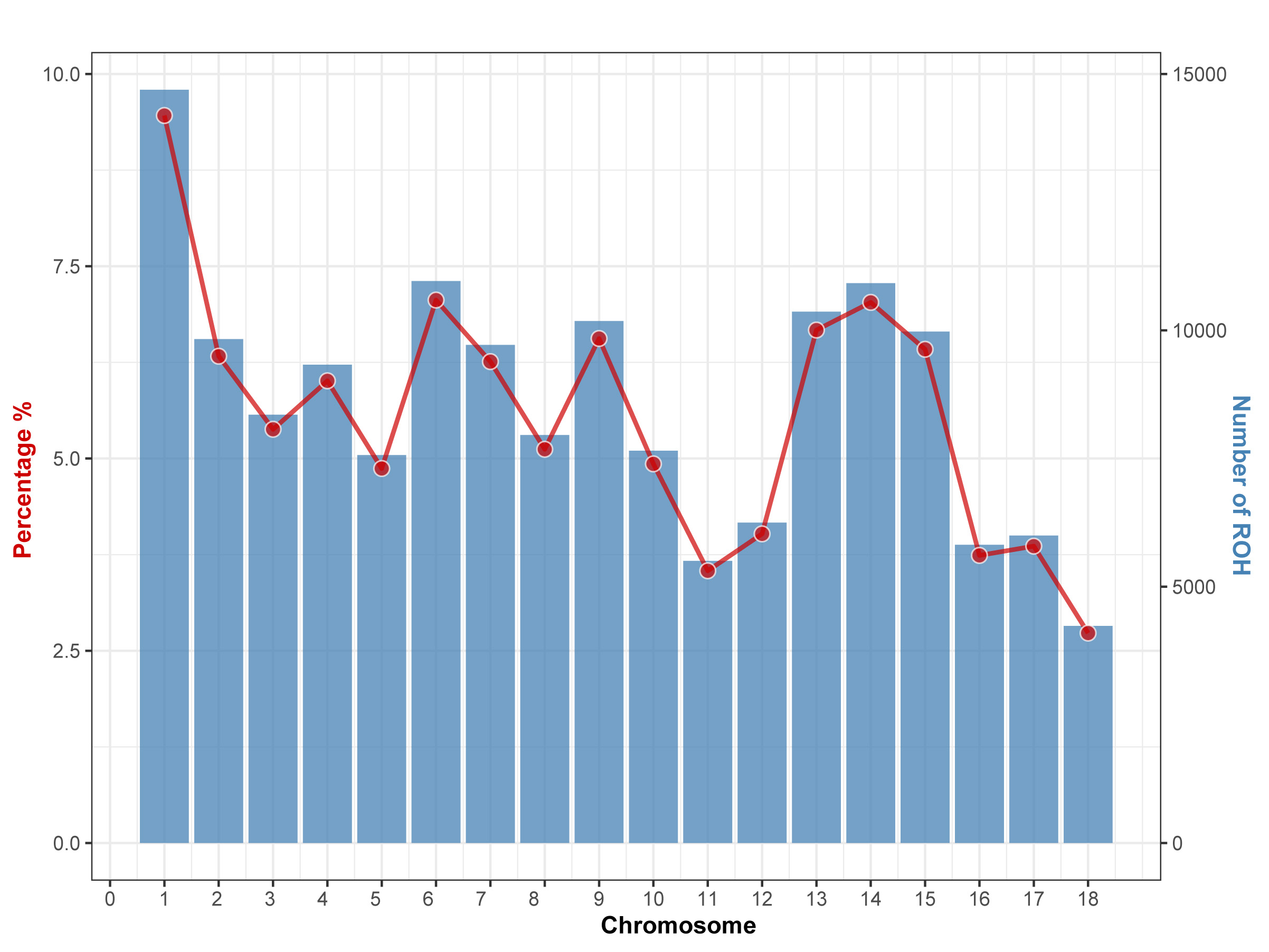

Supplement: Supplementary file 1 [file genes-14-02133-s001.zip › Supplementary Figure1ú║The number and proportion of ROH on each chromosome.jpg]
